# Exploring the global immune landscape of peripheral blood mononuclear cells in H5N6-infected patient with single-cell transcriptomics

**DOI:** 10.1186/s12920-023-01693-7

**Published:** 2023-10-18

**Authors:** Jiamin Gao, Jing Wei, Simei Qin, Sheng Liu, Shuangyan Mo, Qian Long, Shiji Tan, Ning Lu, Zhouhua Xie, Jianyan Lin

**Affiliations:** 1 Laboratory of Infectious Disease, HIV/AIDS Clinical Treatment Center of Guangxi (Nanning), The Fourth People’s Hospital of Nanning, Guangxi Zhuang Autonomous Region, Nanning, 530023 China; 2 Department of Intensive Care Unit, HIV/AIDS Clinical Treatment Center of Guangxi (Nanning) and The Fourth People’s Hospital of Nanning, Nanning, 530023 Guangxi Zhuang Autonomous Region China; 3 Department of Pediatrics, HIV/AIDS Clinical Treatment Center of Guangxi (Nanning) and The Fourth People’s Hospital of Nanning, Guangxi Zhuang Autonomous Region, Nanning, 530023 China; 4 Department of Clinical Laboratory, HIV/AIDS Clinical Treatment Center of Guangxi (Nanning), The Fourth People’s Hospital of Nanning, Nanning, 530023 Guangxi Zhuang Autonomous Region China; 5 Department of Tuberculosis, HIV/AIDS Clinical Treatment Center of Guangxi (Nanning), The Fourth People’s Hospital of Nanning, Guangxi Zhuang Autonomous Region, Nanning, 530023 China

**Keywords:** Avian influenza virus, H5N6, Single cell RNA sequencing, Oxidative stress, Cell death, Immune cells

## Abstract

**Background:**

Avian influenza viruses (AIV), particularly H5N6, have risen in infection frequency, prompting major concerns. Single-cell RNA sequencing (scRNA-seq) can illustrate the immune cell landscape present in the peripheral circulation of influenza H5N6-infected individuals at the single-cell level. This study attempted to employ scRNA-seq technology to map the potentially hidden single cell landscape of influenza H5N6.

**Methods:**

High-quality transcriptomes were generated from scRNA-seq data of peripheral blood mononuclear cells (PBMCs), which were taken from a critically-ill child diagnosed with H5N6 avian influenza infection and one healthy control donor. Cluster analysis was then performed on the scRNA-seq data to identify the different cell types. The pathways, pseudotime developmental trajectories and gene regulatory networks involved in different cell subpopulations were also explored.

**Results:**

In total, 3,248 single cell transcriptomes were captured by scRNA-seq from PBMC of the child infected with H5N6 avian influenza and the healthy control donor and further identified seven immune microenvironment cell types. In addition, a subsequent subpopulation analysis of innate lymphoid cells (ILC) and CD4^+^ T cells revealed that subpopulations of ILC and CD4^+^ T cells were involved in cytokine and inflammation-related pathways and had significant involvement in the biological processes of oxidative stress and cell death.

**Conclusion:**

In conclusion, characterizing the overall immune cell composition of H5N6-infected individuals by assessing the immune cell landscape in the peripheral circulation of H5N6 avian influenza-infected and healthy control donors at single-cell resolution provides key information for understanding H5N6 pathogenesis.

**Supplementary Information:**

The online version contains supplementary material available at 10.1186/s12920-023-01693-7.

## Introduction

Avian influenza virus (AIV) is highly infectious and poses a major public health risk due to its high mortality rate, especially since live bird trade has led to the persistence of many AIV subtypes [1–3]. Recently, the AIV H5Ny spectrum (i.e., H5N1, H5N2, H5N6, and H5N8) has garnered widespread attention due to its transmission ability and genomic rearrangement capabilities between humans and other animal species, which could lead to cross-species infection [4, 5].

In May 2014, the first human became infected with the highly pathogenic H5N6 subtype (evolutionary branch 2.3.4.4), which was reported in China and was the first known human case of H5N6 subtype in the world [6, 7]. This specific H5N6 subtype could bind avian- and human-derived sialic acid receptors and attach to human tracheal epithelium and alveolar tissue, suggesting that it may be a significant public health risk [8, 9]. As of February 18, 2022, a total of 72 human H5N6 cases and 30 deaths have been reported to the World Health Organization in the Western Pacific (WHO Avian Influenza Weekly Update Number 832), with a mortality rate of approximately 42%. However, only a few number of infections have been reported in the pediatric population [10]. The complications of human infection with AIV are mainly respiratory in nature, including respiratory failure, acute respiratory distress syndrome, severe pneumonia, and pulmonary fibrosis during recovery [11, 12]. Once the AIV enters the respiratory epithelium, the innate immune sensor detects the virus, which rapidly initiates a downstream antiviral signaling cascade that leads to the production of multiple cytokines by infected epithelial cells and specific innate immune cells that help limit virus replication and spread. In turn, this leads to the recruitment of activated CD8^+^ T cells, natural killer T (NKT) cells, innate lymphoid cell 2 (ILC2), regulatory T cells (Treg), and T helper cell 2 (Th2) effector cells to move from the capillaries to the site of inflammation [13–16]. Determining the functions and roles among immune cells can identify strategies that may help improve outcomes following H5N6 infection in order to coordinate the immune response.

To this end, this study explores the potential ecological panorama of H5N6-infected individuals via single-cell RNA sequencing (scRNA-seq) of peripheral blood mononuclear cells (PBMCs) from a critically ill child infected with H5N6 avian influenza and a healthy control donor, providing the first single-cell-level insights of H5N6.

## Methods

### Sample source

PBMC samples were obtained from a critically-ill child diagnosed with H5N6 avian influenza infection and a healthy control donor. This study was approved by the Ethics Committee of the Fourth People’s Hospital of Nanning ([2022]03). All procedures involving human participants complied with the ethical standards of the research committee. Informed consent was obtained from participants or their guardians for all study procedures and sequencing protocols.

### scRNA-seq library construction and sequencing

scRNA-seq library construction and sequencing referenced to Liang Y et al [17]. scRNA-seq libraries were prepared using the SeekOne® MM Single Cell 3’ library preparation kit (SeekGene). Briefly, an appropriate number of cells were loaded into the flow channel of the SeekOne® MM chip that contained 170,000 microwells, which was then allowed to settle in the microwells by gravity. After removing the unsettled cells, a sufficient amount of Cell Barcoded Magnetic Beads (CBBs) was pipetted into the flow channel and was also allowed to settle in the microwells with the help of a magnetic field. Next, excess CBBs were rinsed out, and cells in the MM chip were lysed in order to release RNA, which was then captured by the CBB in the same microwell. Then, all CBBs were collected, and reverse transcription was performed at 37℃ for 30 min to label cDNA with cell barcodes on the beads. Further Exonuclease I treatment was performed so as to remove unused primer on the CBBs. Subsequently, barcoded cDNA on the CBBs was hybridized with a random primer that had reads 2 SeqPrimer sequence on the 5’ end and could extend to form the second strand DNA with a cell barcode on the 3’ end. The resulting second strand DNA was denatured off the CBBs, purified and amplified in a Polymerase Chain Reaction (PCR) reaction. The amplified cDNA product was then cleaned to remove unwanted fragments, after which it was added to a full-length sequencing adapter and sample index by indexed PCR. The indexed sequencing libraries were then cleaned with SPRI beads, quantified by quantitative PCR (KAPA Biosystems KK4824) and sequenced on Illumina NovaSeq 6000 with a PE150 read length or DNBSEQ-T7 platform with a PE100 read length. All unique gene names of the transcripts were recorded, the cells were labeled by the barcode, and the transcripts were labeled by unique molecular identifers (UMIs) to quantify the number of cells and genes after comparison with the reference. All reads that mapped to the same gene and had the same UMI sequence were folded and diferent UMIs corresponding to the same gene were quantifed, which produced a digital matrix for cell gene expression quantifcation. For all downstream analyses, we selected cells that have at least 1000 UMIs (indicating the number of captured transcripts) mapped to at least 200 unique genes and ensured that each gene is expressed in more than three cells. We excluded cells with poor viability and quality by removing more than 10% of the cells whose gene counts refected mitochondrial genes or ribosomal RNA (Supplementary Fig. 1).

### Clustering of scRNA-seq data and construction of atlas

Clustering and visualization were performed using the Seurat package in R [18], which annotates clusters using known marker genes that overlap in cluster-specific genes. The “FindAllMarker” function was used for differential expression analysis. In terms of Seurat clustering of raw expression data from filtered cells, annotations were generated using SingleR with the default parameters [19]. The clustering results were then uniformly downscaled and visualized using the Uniform Manifold Approximation and Projection (UMAP) algorithm [20]. In addition, the identified cells were sub-clustered with the Seurat package in order to identify marker genes expressed in each cell sub-cluster using the FindAllMarker function. Finally, cell subpopulations were classified according to the most abundantly expressed marker genes.

### Functional enrichment analysis

In order to determine the potential function of the molecular pathways present in each cell subpopulation, the ClusterProfiler package in R was used for the enrichment analysis [21]. Pathways were considered to be significantly associated with marker genes when P < 0.05.

### Pseudotime analysis

A pseudotime trajectory analysis of cell subpopulations was conducted using the R package Monocle3 [22], which shows the pseudotime changes of cell subpopulations via UMAP plots.

### Gene regulatory network

Single-cell regulatory network inference and clustering (SCENIC) was used to infer gene regulatory networks and identify cellular states based on single-cell expression profiles, which provides an important biological perspective on the mechanisms driving cellular heterogeneity. To identify internal transcriptional regulatory drivers in adipose tissue of different anatomical site origins, gene regulatory networks centered on transcription factors (TFs) were analyzed and reconstructed using the Python module tool pySCENIC [23, 24].

### Data analysis and statistics

All statistical analyses were performed in the Bioinforcloud platform (http://www.bioinforcloud.org.cn), which was applied by calling the appropriate R package (R version 4.0.5). Comparisons between the two groups were made using Student’s t test and correlation coefficients were calculated using Spearman analysis. P < 0.05 was considered significant.

## Results

### The immune global landscape of PBMC in H5N6-infected patient

In the current study, we sought to gain more insight into the immune response to the H5N6 AIV after infection of the host. Accordingly, scRNA-seq of PBMC from an H5N6-infected patient and healthy control donor was performed. The potential ecological panoply of H5N6 using single-cell genomics was then explored using the obtained data.

**Fig. 1:**
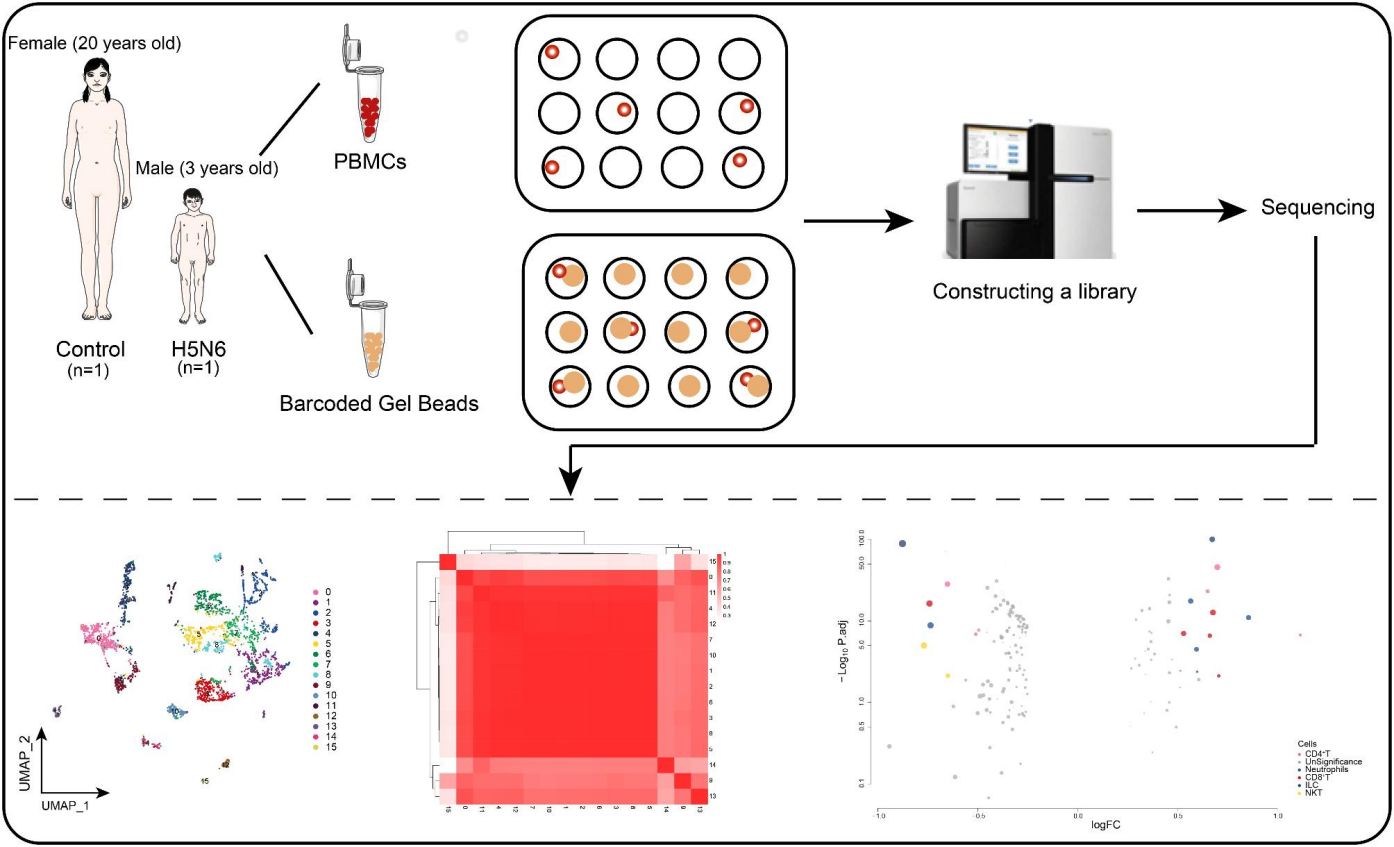
**Workflow of the study.** Single-cell RNA sequencing of PBMCs from an H5N6-infected patient and a healthy control donor captured 16 cell populations that could be used for subsequent analysis. The correlation between cell populations is shown in the heatmap; in addition, differential gene expression analysis was performed to obtain differentially expressed markers for different cell populations. PBMCs, Peripheral blood mononuclear cells; UMAP, Uniform Manifold Approximation and Projection; ILC, Innate lymphoid cell; NKT, Natural killer T

**Fig. 2:**
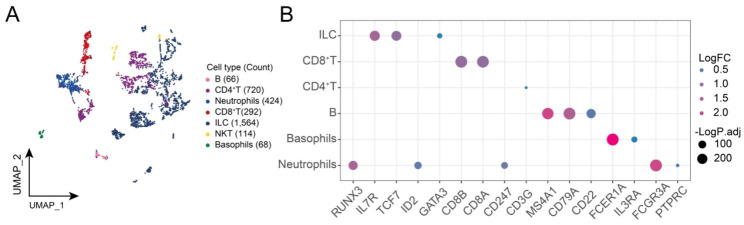
**Global single-cell atlas of H5N6-infected individuals and healthy controls.** (**A**) Single-cell atlas showing the global single-cell landscape of H5N6-infected individuals and healthy controls. (**B**) Bubble plots showing cell-specific markers guiding cell annotation. UMAP, Uniform Manifold Approximation and Projection; ILC, Innate lymphoid cell; NKT, Natural killer T; LogFC, log fold-change

The workflow of this study is shown in Fig. 1. After standardized data processing and quality control, a total of 16 different cell clusters through a clustering analysis. Further identification of cell clusters yielded seven cell types, including B cells, CD8^+^ T cells, CD4^+^ T cells, neutrophils, innate lymphoid cells (ILC), natural killer T cells (NKT) cells and basophils (Fig. 2A), consistent with the established phenotypic characteristics of immune cells (Fig. 2B). In summary, the PBMC single cell atlas of the H5N6-infected and control donors in this study was initially mapped.

### The ILC subpopulation landscape of H5N6

**Fig. 3:**
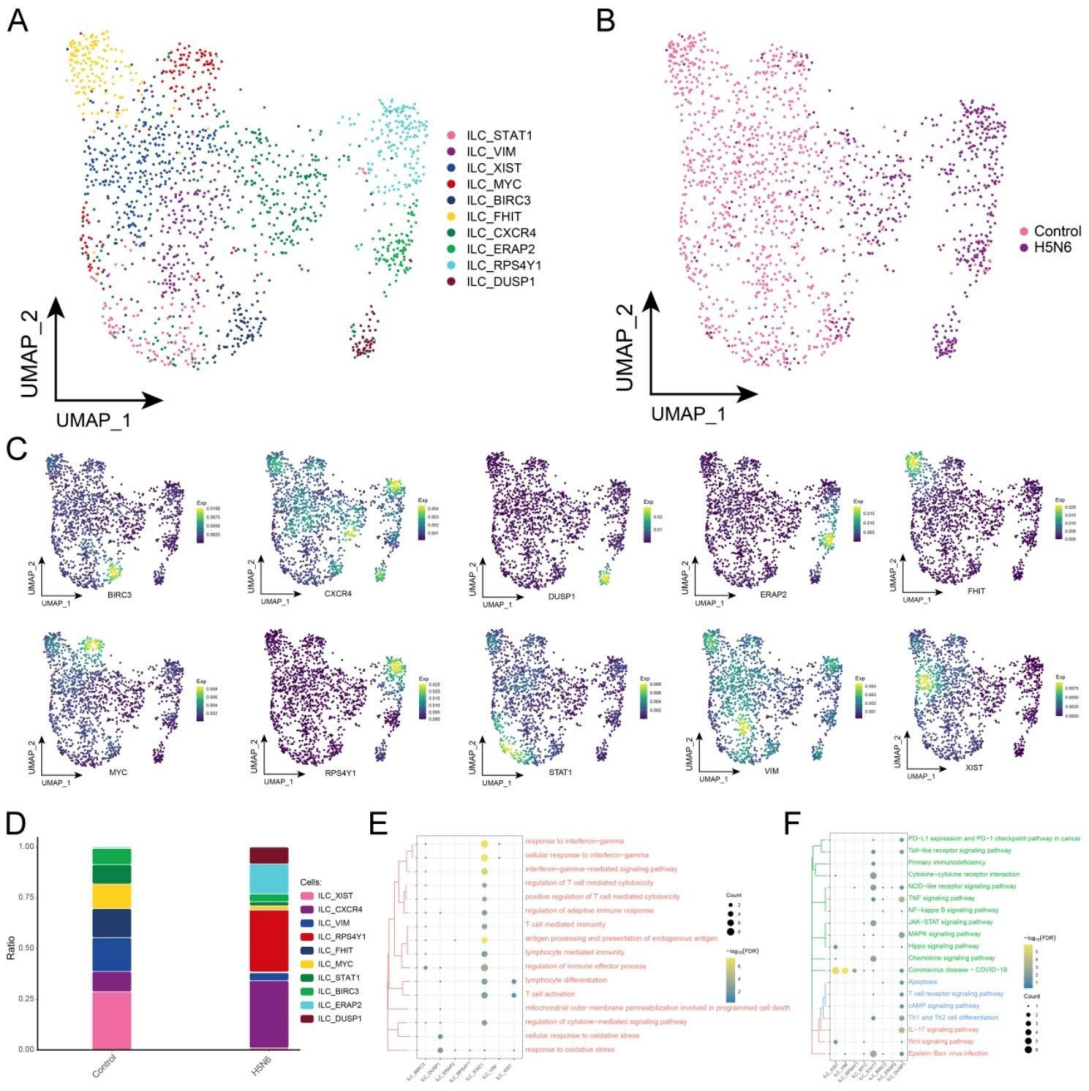
**H5N6-associated ILC subpopulations.** (**A**) Single-cell atlas depicting the ILC subpopulations. (**B**) Single-cell atlas showing the distribution of ILC subpopulations in control-H5N6-infected individuals. (**C**) Series of single-cell atlases illustrating the markers specific to ILC subpopulations. (**D**) The differences in abundance of ILC subpopulations in control and H5N6-infected individuals. (**E**) Biological processes enriched by ILC subpopulations. (**F**) Signaling pathways enriched by ILC subpopulations. UMAP, Uniform Manifold Approximation and Projection; ILC, Innate lymphoid cell; Exp, Expression; FDR, FalseDiscovery Rate

As an intrinsic immune cell, cytokine release, pathogen threat and even tissue homeostatic imbalance can rapidly activate the ILC cell population to function as an early response effector cell in the immune response [25]. In addition, ILCs are capable of both resisting pathogen infection and exerting a protective effect triggering on the organism, and they may also trigger chronic inflammatory diseases [26–28]. Based on cellular ecological mapping at a single-cell resolution, the subpopulations of ILC were explored in-depth, for which 10 subpopulations of ILC were identified (Fig. 3A) that were heterogeneous in H5N6 and Control (Fig. 3B) while mapping the specific markers of each subpopulation in single-cell mapping (Fig. 3C). Among these ILC cell subsets, ILC_CXCR4, ILC_RPS4Y1, ILC_ERAP2, and ILC_DUSP1 subpopulations were found to possess the highest proportion of H5N6-infected individuals (Fig. 3D, Supplementary Table 1). Notably, these subsets were significantly enriched in oxidative stress-related and immuno-related biological processes (Fig. 3E), as well as in *coronavirus disease-COVID-19*, *NOD-like receptor signaling pathway*, *TNF signaling pathway*, *IL-17 signaling pathway*, *Epstein-Barr virus infection* and *PD − L1 expression and PD − 1 checkpoint pathway in cancer* (Fig. 3F).

**Fig. 4:**
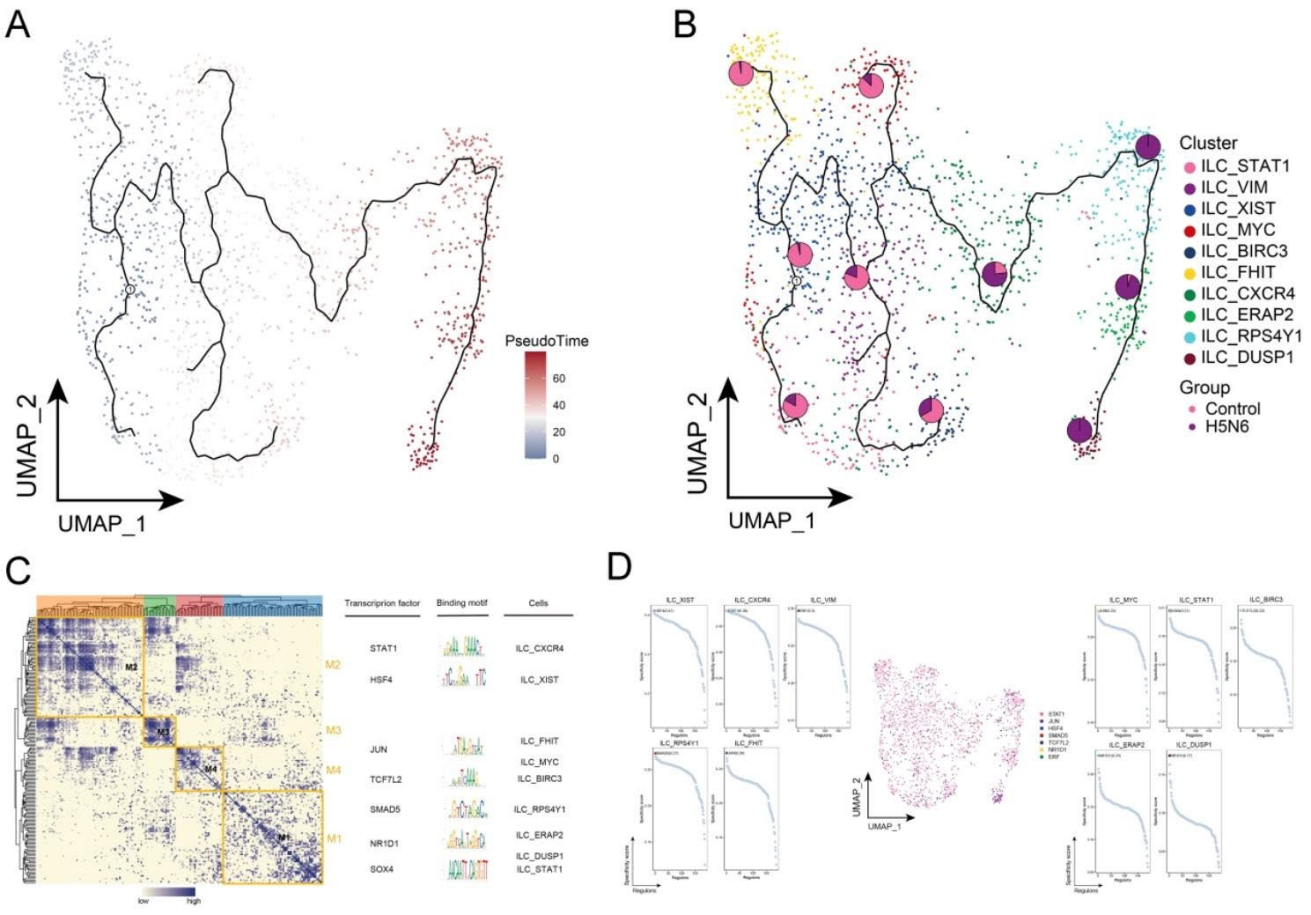
**Cellular developmental trajectories and gene regulatory networks of H5N6-associated ILC subpopulations.** (**A**) Pseudotime analysis showing pseudotime values and pseudotime trajectories of ILC subpopulations from control to disease progression. (**B**) Overlayed pie charts representing the proportion of control and H5N6-infected individuals in each subpopulation. (**C**) Co-expression modules of transcription factors in ILC subpopulations. Left: Identification of regulator modules based on the connection specificity index matrix of regulators. Middle: representative transcription factors and their binding patterns in the modules. Right panel: cell subpopulations in which transcription factors are located. (**D**) Series of scatter plots showing transcription factors regulating ILC subpopulations. UMAP, Uniform Manifold Approximation and Projection; ILC, Innate lymphoid cell

As shown by the pseudotime trajectory analysis, the ILC_XIST subpopulation was noted to be predominantly present in healthy controls, whereas the ILC_RPS4Y1, ILC_ERAP2 and ILC_DUSP1 subpopulations were present in H5N6 patients (Fig. 4A-B). The pseudotime trajectory of cell development reveals the process of ILC subpopulation changes from control donors to H5N6-infected individuals, with the ILC_DUSP1 subpopulation at the end of the pseudotime trajectory. The pseudotime trajectory is consistent with the physiological changes in cell differentiation. Furthermore, gene regulatory networks (GRNs) of ILC cell subpopulations were constructed, and the regulatory factors were hierarchically clustered according to the connectivity specificity index (CSI) in order to rank the importance of regulatory factors and mitigate the effects of non-specific interactions. In doing so, TFs with STAT1, HSF4, JUN, and TCF7L2 were obtained as hubs and were organized into four modules (Fig. 4C). In ILC, cellular reprogramming may promote cellular alterations through GRN driven by these active TFs, which may serve as key TFs involved in the altered cellular state of ILC. In turn, these modules had a significant impact on the regulation of specific gene expression of ILC in H5N6 (Fig. 4D). These findings demonstrate the existence of multiple ILC subpopulations in non-H5N6-infected individuals as well as their heterogeneity. Furthermore, this shows that the inflammatory response pathways involved in ILC subpopulations warrant attention.

### CD4^+^ T cell subpopulation landscape in H5N6 infected patient

**Fig. 5:**
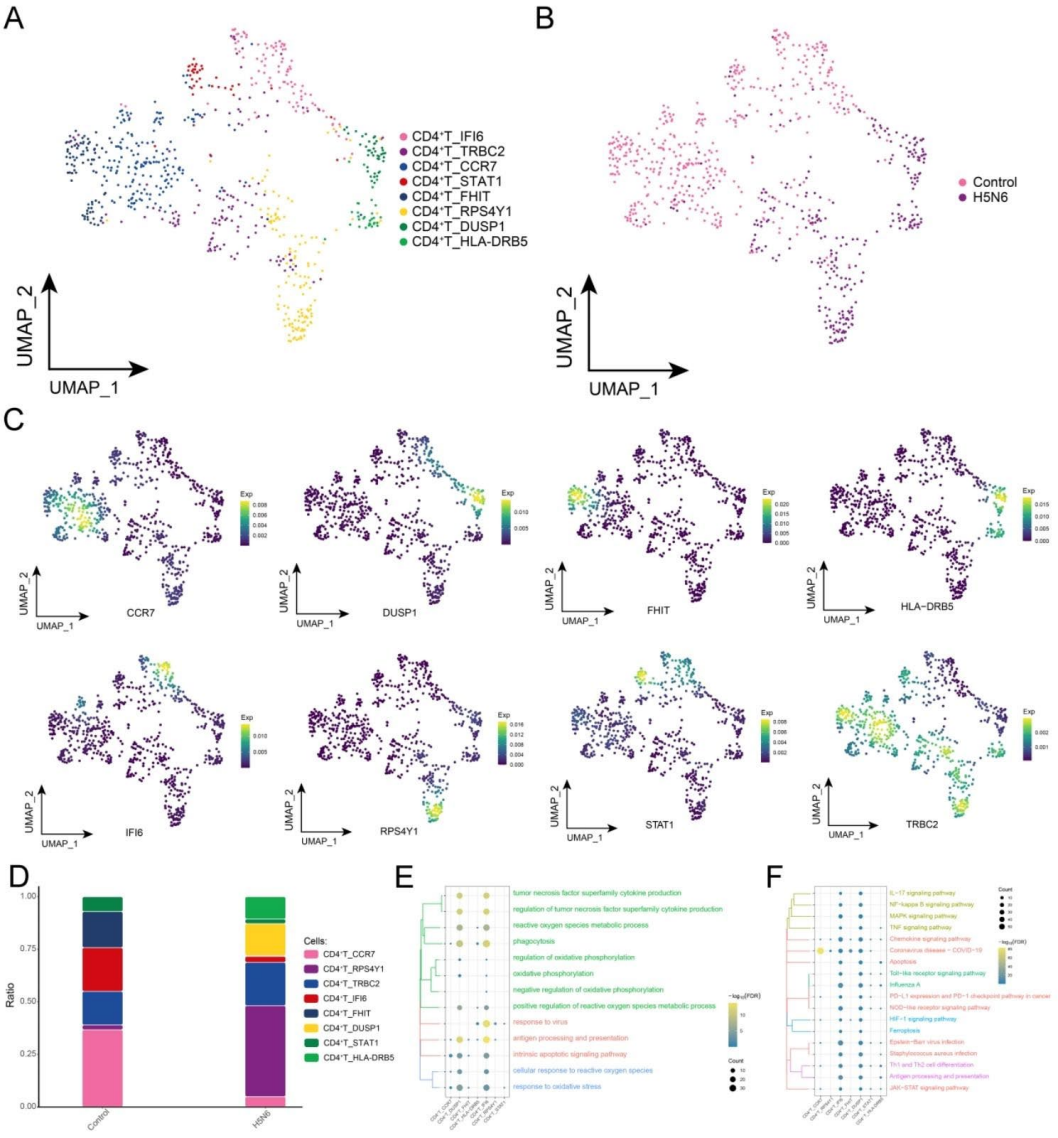
**H5N6-associated CD4**^**+**^**T cell subpopulations.** (**A**) Single-cell atlas showing CD4^+^ T cell subpopulations. (**B**) Single-cell atlas illustrating the distribution of CD4^+^ T cell subpopulations in control and H5N6-infected individuals. (**C**) Series of single-cell atlases depicting the markers specific for CD4^+^ T cell subpopulations. (**D**) The difference in abundance of CD4^+^ T cell subpopulations in control and H5N6-infected individuals. (**E**) Biological processes enriched by CD4^+^ T cell subpopulations. (**F**) Signaling pathways enriched by CD4^+^ T cell subpopulations. UMAP, Uniform Manifold Approximation and Projection; Exp, Expression; FDR, FalseDiscovery Rate

AIV infection can lead to higher mortality, which has been shown to be closely associated with an excessive inflammatory response [29, 30]. Meanwhile, Th17 differentiation of CD4^+^ T cells has been shown to be involved in mediating inflammatory cell infiltration, leading to severe pathological tissue damage and dysfunction of the host [31, 32]. Eight CD4^+^ T cell subpopulations were identified by subpopulation analysis in this study (Fig. 5A), and these subpopulations were heterogeneous in H5N6 and Control (Fig. 5B), with specific markers being mapped in the single-cell profiles (Fig. 5C). Notably, among these subpopulations of CD4^+^ T cells, CD4^+^ T_RPS4Y1, CD4^+^ T_TRBC2, CD4^+^ T_DUSP1 and CD4^+^ T_HLA-DRB5 subpopulations were found to be significantly abundant among H5N6-infected individuals (Fig. 5D, Supplementary Table 2). These subpopulations were also observed to be significantly enriched in biological processes associated with oxidative stress and cell death (Fig. 5E). Specifically, the Kyoto Encyclopedia of Genes and Genomes (KEGG) signaling pathway analysis showed that *coronavirus disease-COVID-19*, *chemokine signaling pathway*, *TNF signaling pathway*, *IL-17 signaling pathway*, *influenza A* and *PD − L1 expression and PD − 1 checkpoint pathway in cancer* were enriched (Fig. 5F) [33–35].

**Fig. 6:**
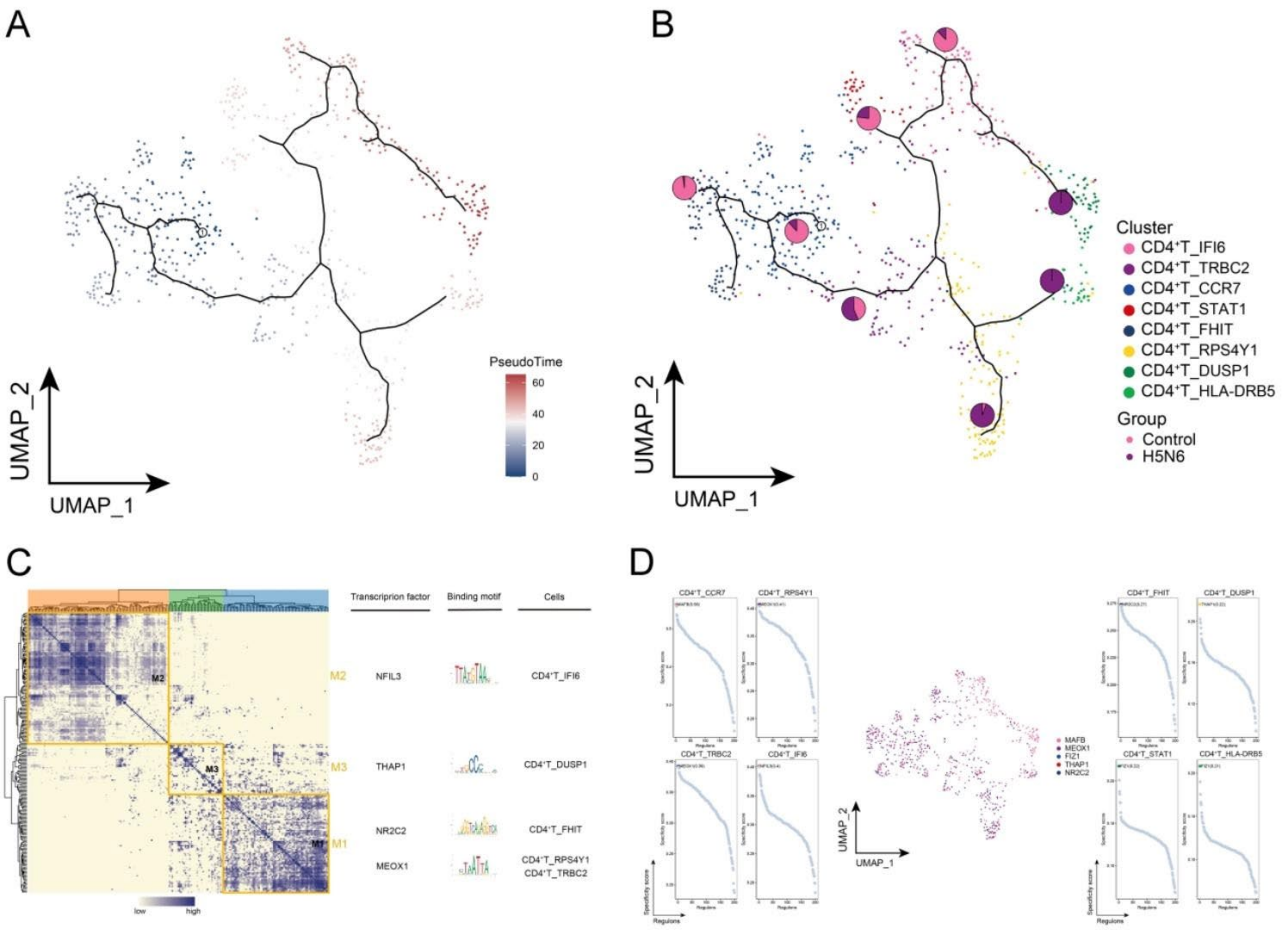
**Cellular developmental trajectories and gene regulatory networks of H5N6-associated CD4**^**+**^**T cell subpopulations.** (**A**) Pseudotime analysis demonstrating pseudotime values and pseudotime trajectories of CD4^+^ T cell subpopulations from control to disease progression. (**B**) Overlayed pie charts representing the proportion of control and H5N6-infected individuals in each subpopulation. (**C**) Co-expression modules of transcription factors in CD4^+^ T cell subpopulations. Left: identification of regulator modules based on the connection specificity index matrix of regulators. Middle: representative transcription factors and their binding patterns in the modules. Right panel: cell subpopulations where transcription factors are located. (**D**) Series of scatter plots showing transcription factors regulating CD4^+^ T cell subpopulations. UMAP, Uniform Manifold Approximation and Projection

The pseudotime trajectory of CD4^+^ T cell subpopulations were analyzed, in which CD4^+^ T_CCR7 subpopulation was mainly in control donors, while CD4^+^ T_RPS4Y1, CD4^+^ T_DUSP1 and CD4^+^ T_HLA-DRB5 subpopulations were significantly enriched in H5N6 (Fig. 6A-B). The pseudotime trajectories of CD4^+^ T cell subpopulations were analyzed, in which the CD4^+^ T_CCR7 subpopulation was mainly in control donors, while the CD4^+^ T_RPS4Y1, CD4^+^ T_DUSP1 and CD4^+^ T_HLA-DRB5 subpopulations were significantly enriched in H5N6 (Fig. 6A-B). The pseudotime trajectory of cell development revealed the process of CD4^+^ T subpopulation changes from control donors to H5N6-infected individuals, with CD4^+^ T_RPS4Y1, CD4^+^ T_DUSP1 and CD4^+^ T_HLA-DRB5 subpopulations subpopulations at the end of the pseudotime trajectory, and the pseudotime trajectory was consistent with the physiological changes in cell differentiation, making them specific subpopulations in H5N6. According to GRN, CD4^+^ T_IFI6, CD4^+^ T_DUSP1, CD4^+^ T_FHIT, CD4^+^ T_RPS4Y1 and CD4^+^ T_TRBC2 subpopulations were regulated by NFIL3, THAP1, NR2C2 and MEOX1, respectively (Fig. 6C-D). According to GRN, CD4^+^ T cell subpopulations were regulated by NFIL3, THAP1, NR2C2 and MEOX1, respectively (Fig. 6C-D). In CD4^+^ T, cellular reprogramming may promote cellular alterations through GRN driven by these active TFs, which may serve as key TFs involved in the altered cellular state of CD4^+^ T. In conclusion, by identifying subpopulations of CD4^+^ T cells, a subpopulation of CD4^+^ T cells in H5N6 involved in immune and inflammation-related pathways that may contribute to the excessive inflammatory response in H5N6 were identified.

## Discussion

Previous studies have shown that AIV emerge through multiple genetic rearrangements of different subtypes of viruses within populations of resident poultry and wild birds, which may also persist in poultry populations, thereby leading to widespread transmission with pandemic potential [36–38]. In addition, the H5N6 subtype of the AIV can pose a serious threat to human health [39]. Due to its potentially high pathogenicity and cross-species transmissibility, this study attempted to present a comprehensive profile of cell types and subpopulations in PBMC samples from both H5N6-infected and healthy control donors at a single-cell resolution. By assessing the ecological changes and different signaling profiles of subpopulations of different cell types and analyzing the developmental trajectories and transcriptional regulators of different cell subpopulations, the corresponding findings could provide novel insight into transcriptional differences between cells of different origins in H5N6-infected individuals.

Seven independent cell types were initially identified in this analysis, including certain immune cells. Each cell type showed different pathway characteristics and activity between PBMC from H5N6-infected and healthy control donors.

As an important effector cell, ILC has an important role in maintaining tissue homeostasis and its response to tissue injury [40]. As tissue-resident innate immune cells, ILCs can be divided into multiple subpopulations according to their specific functions [41], which may both participate in immunity and cause associated inflammation [42–45]. In addition, ILCs have been shown to be involved in the regulation of immune responses and tissue homeostasis [46]. Despite recent advances in studying ILCs, the cellular heterogeneity, developmental trajectory and functional role of ILC cells in H5N6 have yet to be understood. Accordingly, this study demonstrated the presence of ILC subpopulations in H5N6 and highlighted the developmental stages and pathways of different ILC subpopulations in H5N6. Previous studies have offered insight into the novel functions of murine lung ILCs in regulating airway epithelial barrier integrity and tissue homeostasis following influenza virus-induced lung injury [47]. The present study suggested that ILC subpopulations are significantly heterogeneous, and these cell subpopulations exhibit different developmental requirements and patterns of cellular transcription factor expression. In another study on H5N6, Bi et al. found that humoral and cellular responses were detectable in survivors of H5N6 infection, which were not present among non-survivors. In addition, survivors had lower concentrations of pro- and anti-inflammatory cytokines/chemokines compared to those of non-survivors [48]. These findings were consistent with those of the present study, where the enrichment analysis of ILC subpopulations revealed that ILC subpopulations with specificity were significantly involved in a number of cytokine interactions as well as inflammation-related pathways. In addition, cells in the innate immune system produce ROS, and long-term inflammatory processes can increase ROS production, causing oxidative stress, which was verified in this study as well [49].

Survival and recovery from AIV infection depends on the development of strong T-cell immunity in the host, leading to memory responses, which has been previously observed in H7N9 and pH1N1 infections [50–52]. Interestingly, no reports on CD4^+^ T cell immunity in patients with H5N6 virus infection currently exist. The presence of H5N6 virus-specific T cells observed in this single-cell resolution may play a role in controlling disease progression and viral clearance, thus contributing to survival. In addition, avian influenza vaccines have demonstrated poor immunogenicity and efficacy in humans [53, 54]. One study suggested that the poor antibody response to avian influenza vaccine was due to insufficient help from CD4^+^ T cells [55]. In addition, enriched oxidative stress-related signaling pathways warrant further attention. Inflammation and oxidative stress can adversely affect T cells, and CD4^+^ T cells are important mediators of oxidative stress [56]. Thus, the scRNA-seq data from this study can provide further context for the lack of cellular immune analyses in H5N6.

In conclusion, our study is the first to examine H5N6 avian influenza at single-cell resolution, filling a gap in the understanding of H5N6 avian influenza. scRNA-seq of PBMC samples from H5N6-infected individuals identified immune pathways corresponding to differences in host gene expression throughout infection, thus deepening the understanding of immune processes that occur during infection, which may later help in the development of therapeutic approaches for infected patients. The present study has some limitations. First, the sample size of this study was small, and the results of this study need to be validated with a larger sample of patients, which we will continue to collect in the future to enrich the study. Second, the conclusions obtained from this study are based on bioinformatics analysis, and future cellular and molecular experiments will be needed to validate them.

### Electronic supplementary material

Below is the link to the electronic supplementary material.

Supplementary Material 1

Supplementary Material 2

Supplementary Material 3

## Data Availability

The dataset supporting the conclusions of this article is available in the National Genomics Data Center repository, [(GSA: HRA003053) and https://ngdc.cncb.ac.cn/gsa-human/browse/HRA003053].

## Abbreviations

AIV: Avian influenza viruses
scRNA-seq: Single-cell RNA sequencing
PBMCs: Peripheral blood mononuclear cells
ILC: Innate lymphoid cells
WHO: World Health Organization
NKT: Natural killer T
Treg: Regulatory T cells
Th2: T helper cell 2
CBBs: Cell Barcoded Magnetic Beads
UMAP: Uniform Manifold Approximation and Projection
SCENIC: Single cell regulatory network inference and clustering
GRN: Gene regulatory network
CSI: Connectivity specificity index
KEGG: Kyoto Encyclopedia of Genes and Genomes
PCR: Polymerase Chain Reaction
TF: Transcription factor

## References

[1] Liu D, Shi W, Shi Y, Wang D, Xiao H, Li W (2013). Origin and diversity of novel avian influenza a H7N9 viruses causing human infection: phylogenetic, structural, and coalescent analyses. Lancet.

[2] Gao GF (2014). Influenza and the live poultry trade. Science.

[3] Liu WJ, Wu Y, Bi Y, Shi W, Wang D, Shi Y et al. Emerging HxNy Influenza A Viruses. Cold Spring Harb Perspect Med. 2022;12(2). 10.1101/cshperspect.a038406 PMC8805644 32928891

[4] Shi W, Gao GF (2021). Emerging H5N8 avian influenza viruses. Science.

[5] Quan C, Wang Q, Zhang J, Zhao M, Dai Q, Huang T (2019). Avian influenza a viruses among occupationally exposed populations, China, 2014–2016. Emerg Infect Dis.

[6] Zhang R, Chen T, Ou X, Liu R, Yang Y, Ye W (2016). Clinical, epidemiological and virological characteristics of the first detected human case of avian influenza A(H5N6) virus. Infect Genet Evol.

[7] Pan M, Gao R, Lv Q, Huang S, Zhou Z, Yang L (2016). Human infection with a novel, highly pathogenic avian influenza A (H5N6) virus: virological and clinical findings. J Infect.

[8] Sun H, Pu J, Wei Y, Sun Y, Hu J, Liu L (2016). Highly pathogenic avian influenza H5N6 viruses exhibit enhanced Affinity for Human Type Sialic Acid receptor and In-Contact transmission in Model ferrets. J Virol.

[9] Bi Y, Liu H, Xiong C, Di L, Shi W, Li M (2016). Novel avian influenza A (H5N6) viruses isolated in migratory waterfowl before the first human case reported in China, 2014. Sci Rep.

[10] Chen T, Zhang R (2015). Symptoms seem to be mild in children infected with avian influenza A (H5N6) and other subtypes. J Infect.

[11] Taubenberger JK, Morens DM (2008). The pathology of influenza virus infections. Annu Rev Pathol.

[12] Peiris JS, de Jong MD, Guan Y (2007). Avian influenza virus (H5N1): a threat to human health. Clin Microbiol Rev.

[13] Hartshorn KL (2020). Innate immunity and influenza a Virus Pathogenesis: Lessons for COVID-19. Front Cell Infect Microbiol.

[14] Wang Z, Loh L, Kedzierski L, Kedzierska K (2016). Avian influenza viruses, inflammation, and CD8(+) T cell immunity. Front Immunol.

[15] Mao H, Liu Y, Sia SF, Peiris JSM, Lau YL, Tu W (2017). Avian influenza virus directly infects human natural killer cells and inhibits cell activity. Virol Sin.

[16] Lamichhane PP, Samarasinghe AE (2019). The role of Innate Leukocytes during Influenza Virus infection. J Immunol Res.

[17] Liang Y, He H, Wang W, Wang H, Mo S, Fu R (2022). Malignant clonal evolution drives multiple myeloma cellular ecological diversity and microenvironment reprogramming. Mol Cancer.

[18] Stuart T, Butler A, Hoffman P, Hafemeister C, Papalexi E, Mauck WM (2019). Comprehensive Integration of single-cell data. Cell.

[19] Aran D, Looney AP, Liu L, Wu E, Fong V, Hsu A (2019). Reference-based analysis of lung single-cell sequencing reveals a transitional profibrotic macrophage. Nat Immunol.

[20] Becht E, McInnes L, Healy J, Dutertre CA, Kwok IWH, Ng LG et al. Dimensionality reduction for visualizing single-cell data using UMAP. Nat Biotechnol. 2018. 10.1038/nbt.4314 30531897

[21] Yu G, Wang LG, Han Y, He QY (2012). clusterProfiler: an R package for comparing biological themes among gene clusters. OMICS.

[22] Trapnell C, Cacchiarelli D, Grimsby J, Pokharel P, Li S, Morse M (2014). The dynamics and regulators of cell fate decisions are revealed by pseudotemporal ordering of single cells. Nat Biotechnol.

[23] Aibar S, Gonzalez-Blas CB, Moerman T, Huynh-Thu VA, Imrichova H, Hulselmans G (2017). SCENIC: single-cell regulatory network inference and clustering. Nat Methods.

[24] Van de Sande B, Flerin C, Davie K, De Waegeneer M, Hulselmans G, Aibar S (2020). A scalable SCENIC workflow for single-cell gene regulatory network analysis. Nat Protoc.

[25] Wang X, Peng H, Tian Z (2019). Innate lymphoid cell memory. Cell Mol Immunol.

[26] Nagashima H, Mahlakoiv T, Shih HY, Davis FP, Meylan F, Huang Y (2019). Neuropeptide CGRP limits Group 2 innate lymphoid cell responses and constrains type 2 inflammation. Immunity.

[27] Grigg JB, Shanmugavadivu A, Regen T, Parkhurst CN, Ahmed A, Joseph AM (2021). Antigen-presenting innate lymphoid cells orchestrate neuroinflammation. Nature.

[28] Wu J, Lv X, Zhu S, Li T, Cheng H, Chen J (2019). Critical roles of balanced innate lymphoid cell subsets in intestinal homeostasis, chronic inflammation, and Cancer. J Immunol Res.

[29] Pantin-Jackwood MJ, Swayne DE (2009). Pathogenesis and pathobiology of avian influenza virus infection in birds. Rev Sci Tech.

[30] Myers MA, Smith AP, Lane LC, Moquin DJ, Aogo R, Woolard S et al. Dynamically linking influenza virus infection kinetics, lung injury, inflammation, and disease severity. Elife. 2021;10. 10.7554/eLife.68864 PMC8370774 34282728

[31] Sant AJ, DiPiazza AT, Nayak JL, Rattan A, Richards KA (2018). CD4 T cells in protection from influenza virus: viral antigen specificity and functional potential. Immunol Rev.

[32] Bao J, Cui D, Wang X, Zou Q, Zhao D, Zheng S (2019). Decreased Frequencies of Th17 and Tc17 cells in patients infected with avian influenza A (H7N9) virus. J Immunol Res.

[33] Kanehisa M, Goto S (2000). KEGG: kyoto encyclopedia of genes and genomes. Nucleic Acids Res.

[34] Kanehisa M (2019). Toward understanding the origin and evolution of cellular organisms. Protein Sci.

[35] Kanehisa M, Furumichi M, Sato Y, Kawashima M, Ishiguro-Watanabe M (2023). KEGG for taxonomy-based analysis of pathways and genomes. Nucleic Acids Res.

[36] Bi Y, Li J, Li S, Fu G, Jin T, Zhang C (2020). Dominant subtype switch in avian influenza viruses during 2016–2019 in China. Nat Commun.

[37] Wan XF, Dong L, Lan Y, Long LP, Xu C, Zou S (2011). Indications that live poultry markets are a major source of human H5N1 influenza virus infection in China. J Virol.

[38] Zhang T, Bi Y, Tian H, Li X, Liu D, Wu Y (2014). Human infection with influenza virus A(H10N8) from live poultry markets, China, 2014. Emerg Infect Dis.

[39] Bi Y, Chen Q, Wang Q, Chen J, Jin T, Wong G (2016). Genesis, Evolution and Prevalence of H5N6 Avian Influenza Viruses in China. Cell Host Microbe.

[40] De Pasquale C, Campana S, Bonaccorsi I, Carrega P, Ferlazzo G (2021). ILC in chronic inflammation, cancer and targeting with biologicals. Mol Aspects Med.

[41] Vivier E, Artis D, Colonna M, Diefenbach A, Di Santo JP, Eberl G (2018). Innate lymphoid cells: 10 years on. Cell.

[42] Chiossone L, Dumas PY, Vienne M, Vivier E (2018). Natural killer cells and other innate lymphoid cells in cancer. Nat Rev Immunol.

[43] Bie Q, Zhang P, Su Z, Zheng D, Ying X, Wu Y (2014). Polarization of ILC2s in peripheral blood might contribute to immunosuppressive microenvironment in patients with gastric cancer. J Immunol Res.

[44] Chan IH, Jain R, Tessmer MS, Gorman D, Mangadu R, Sathe M (2014). Interleukin-23 is sufficient to induce rapid de novo gut tumorigenesis, independent of carcinogens, through activation of innate lymphoid cells. Mucosal Immunol.

[45] Kirchberger S, Royston DJ, Boulard O, Thornton E, Franchini F, Szabady RL (2013). Innate lymphoid cells sustain colon cancer through production of interleukin-22 in a mouse model. J Exp Med.

[46] Yu Y, Tsang JC, Wang C, Clare S, Wang J, Chen X (2016). Single-cell RNA-seq identifies a PD-1(hi) ILC progenitor and defines its development pathway. Nature.

[47] Monticelli LA, Sonnenberg GF, Abt MC, Alenghat T, Ziegler CG, Doering TA (2011). Innate lymphoid cells promote lung-tissue homeostasis after infection with influenza virus. Nat Immunol.

[48] Bi Y, Tan S, Yang Y, Wong G, Zhao M, Zhang Q (2019). Clinical and immunological characteristics of human infections with H5N6 avian influenza virus. Clin Infect Dis.

[49] Agita A, Alsagaff MT (2017). Inflammation, immunity, and hypertension. Acta Med Indones.

[50] Wang Z, Wan Y, Qiu C, Quinones-Parra S, Zhu Z, Loh L (2015). Recovery from severe H7N9 disease is associated with diverse response mechanisms dominated by CD8(+) T cells. Nat Commun.

[51] Hung IF, To KK, Lee CK, Lin CK, Chan JF, Tse H (2010). Effect of clinical and virological parameters on the level of neutralizing antibody against pandemic influenza a virus H1N1 2009. Clin Infect Dis.

[52] Henry Dunand CJ, Leon PE, Huang M, Choi A, Chromikova V, Ho IY (2016). Both neutralizing and non-neutralizing human H7N9 Influenza Vaccine-Induced Monoclonal antibodies Confer Protection. Cell Host Microbe.

[53] Nichol KL, Treanor JJ (2006). Vaccines for seasonal and pandemic influenza. J Infect Dis.

[54] de Vries RD, Herfst S, Richard M. Avian influenza a virus pandemic preparedness and Vaccine Development. Vaccines (Basel). 2018;6(3). 10.3390/vaccines6030046 PMC6161001 30044370

[55] DiPiazza AT, Fan S, Rattan A, DeDiego ML, Chaves F, Neumann G (2019). A Novel Vaccine Strategy to Overcome Poor Immunogenicity of Avian Influenza Vaccines through mobilization of memory CD4 T cells established by Seasonal Influenza. J Immunol.

[56] Ivanov AV, Valuev-Elliston VT, Ivanova ON, Kochetkov SN, Starodubova ES, Bartosch B (2016). Oxidative stress during HIV infection: mechanisms and consequences. Oxid Med Cell Longev.

