# Supplementary material for: Exploring the global immune landscape of peripheral blood mononuclear cells in H5N6-infected patient with single-cell transcriptomics
Source: BMC Med Genomics. 2023 Oct 18;16:249. doi: 10.1186/s12920-023-01693-7 (PMC10585775; doi:10.1186/s12920-023-01693-7)

Supplementary Figure 1. Quality Control

Number of double cells, dead cells and mitochondrial genes before and after quality control.

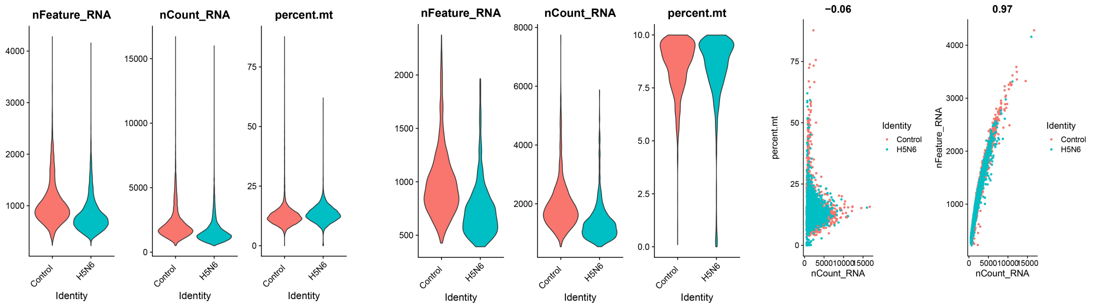

Supplement: Supplementary file 1 — Supplementary Material 1 [file 12920_2023_1693_MOESM1_ESM.pdf]
