# Supplementary material for: Exploring the global immune landscape of peripheral blood mononuclear cells in H5N6-infected patient with single-cell transcriptomics
Source: BMC Med Genomics. 2023 Oct 18;16:249. doi: 10.1186/s12920-023-01693-7 (PMC10585775; doi:10.1186/s12920-023-01693-7)
Supplement: Supplementary file 2 — Supplementary Material 2 [file 12920_2023_1693_MOESM2_ESM.pdf]

Supplementary Table 1. The difference in abundance of ILC subpopulations in control and H5N6-infected individuals.

BarCol	Group	sum(Ratio)
ILC_STAT1	Control	0.288
ILC_STAT1	H5N6	0.010638298
ILC_VIM	Control	0.099
ILC_VIM	H5N6	0.331560284
ILC_XIST	Control	0.167
ILC_XIST	H5N6	0.039007092
ILC_MYC	Control	0
ILC_MYC	H5N6	0.308510638
ILC_BIRC3	Control	0.143
ILC_BIRC3	H5N6	0.003546099
ILC_FHIT	Control	0.121
ILC_FHIT	H5N6	0.017730496
ILC_CXCR4	Control	0.096
ILC_CXCR4	H5N6	0.019503546
ILC_ERAP2	Control	0.079
ILC_ERAP2	H5N6	0.039007092
ILC_RPS4Y1	Control	0.007
ILC_RPS4Y1	H5N6	0.147163121
ILC_DUSP1	Control	0
ILC_DUSP1	H5N6	0.083333333
