# Supplementary material for: Exploring the global immune landscape of peripheral blood mononuclear cells in H5N6-infected patient with single-cell transcriptomics
Source: BMC Med Genomics. 2023 Oct 18;16:249. doi: 10.1186/s12920-023-01693-7 (PMC10585775; doi:10.1186/s12920-023-01693-7)
Supplement: Supplementary file 3 — Supplementary Material 3 [file 12920_2023_1693_MOESM3_ESM.pdf]

Supplementary Table 2. The difference in abundance of CD4+ T cell subpopulations in control and H5N6-infected individuals.

BarCol	Group	sum(Ratio)
CD4.T_IFI6	Control	0.367579909
CD4.T_IFI6	H5N6	0.04964539
CD4.T_TRBC2	Control	0.02283105
CD4.T_TRBC2	H5N6	0.432624113
CD4.T_CCR7	Control	0.159817352
CD4.T_CCR7	H5N6	0.205673759
CD4.T_STAT1	Control	0.207762557
CD4.T_STAT1	H5N6	0.028368794
CD4.T_FHIT	Control	0.171232877
CD4.T_FHIT	H5N6	0.003546099
CD4.T_RPS4Y1	Control	0
CD4.T_RPS4Y1	H5N6	0.15248227
CD4.T_DUSP1	Control	0.070776256
CD4.T_DUSP1	H5N6	0.021276596
CD4.T_HLA-DRB5	Control	0
CD4.T_HLA-DRB5	H5N6	0.106382979
